# Maxillary Odontoma Associated With Noonan Syndrome: A Case Report

**DOI:** 10.7759/cureus.52699

**Published:** 2024-01-21

**Authors:** Hussam Z Alsalem, Munira Alshahrani, Bader Fatani, Ali A Alshehri, Rana M Almutairi, Raghad F Almuqrin

**Affiliations:** 1 Oral and Maxillofacial Surgery, Prince Sultan Military Medical City, Ministry of Defense and Aviation, Riyadh, SAU; 2 Oral and Maxillofacial Surgery, King Khalid University, Abha, SAU; 3 Dentistry, King Saud University, College of Dentistry, Riyadh, SAU; 4 Dentistry, King Khalid University, Abha, SAU; 5 Dentistry, King Saud Bin Abdulaziz University for Health Sciences, Riyadh, SAU

**Keywords:** maxillofacial surgery, supernumerary teeth, maxillary odontoma, odontoma, noonan syndrome

## Abstract

Noonan syndrome (NS) is a common congenital syndrome characterized by multiple anomalies commonly observed in children. In this article, we describe a case of a patient with congenital heart disease, severe mitral regurgitation, and Nonaan syndrome presented with left maxillary swelling and pain, which was treated by complete surgical excision of the left maxillary odontoma. Based on this case, we conclude that numerous oral abnormalities may be related to NS and thus necessitate interdisciplinary treatment planning and prompt therapy. The importance of including oral manifestations as a scoring criterion in diagnosing NS cannot be overstated, as the significance of oral findings in NS has largely been overlooked.

## Introduction

Noonan syndrome (NS) is a common congenital syndrome characterized by multiple anomalies commonly observed in children. Its occurrence has been estimated to range from one in 1,000 to one in 2,500 individuals. An early illustration of this syndrome was done by Jacqueline Noonan identified distinctive facial features, congenital heart defects, and other manifestations. Initially referred to as "male Turner syndrome" due to similarities with Turner syndrome, it has since been recognized that NS can affect both genders and typically involves normal chromosomes. NS is the second most common cause of congenital heart disease associated with specific symptoms, such as hypertrophic cardiomyopathy and valvular pulmonary stenosis [1]. NS was initially identified in 1963 by Jacqueline Noonan. She documented the occurrence of valvular pulmonary stenosis in nine children who displayed a phenotype resembling Turner syndrome. Remarkably, these children had a normal karyotype and could be either male or female [2]. This syndrome is characterized by various oral manifestations, such as a high-arched palate, misalignment of the teeth, abnormally small jaw, abnormalities in the number of teeth, cysts in the jaw resembling cherubism, severe tooth decay, gum diseases, and large cell lesions [3]. The syndrome often comes with common associations, such as a neck with skin folds, abnormalities in the chest, intellectual disability, and problems with bleeding [4].

The dental care of patients with NS, especially those with heart defects or bleeding disorders, should involve collaboration among the patient's dentist, general medical practitioner, pediatrician, cardiologist, hematologist, and dental specialist. It is crucial to emphasize the significance of regular dental visits, preventive measures such as oral hygiene and dietary advice, fluoride, fissure sealing, and prompt treatment of dental decay [5]. Because gingivitis, dental decay, and misalignment of the teeth are more common in NS patients, it is recommended that a comprehensive approach, including regular dental check-ups, should be the standard care for these individuals [6].

The literature does not thoroughly describe the oral signs of NS. However, certain features, such as a high-arched palate, small jaw, enlarged teeth, underdeveloped tooth enamel, missing teeth, and an abnormal shape of the tooth roots, have consistently been linked to NS [7]. To this day, there is not enough information in the literature regarding the occurrence of odontoma in patients with NS. This case report demonstrates a maxillary odontoma in a patient with NS who was fully treated under general anesthesia (GA).

## Case presentation

**Figure 1:**
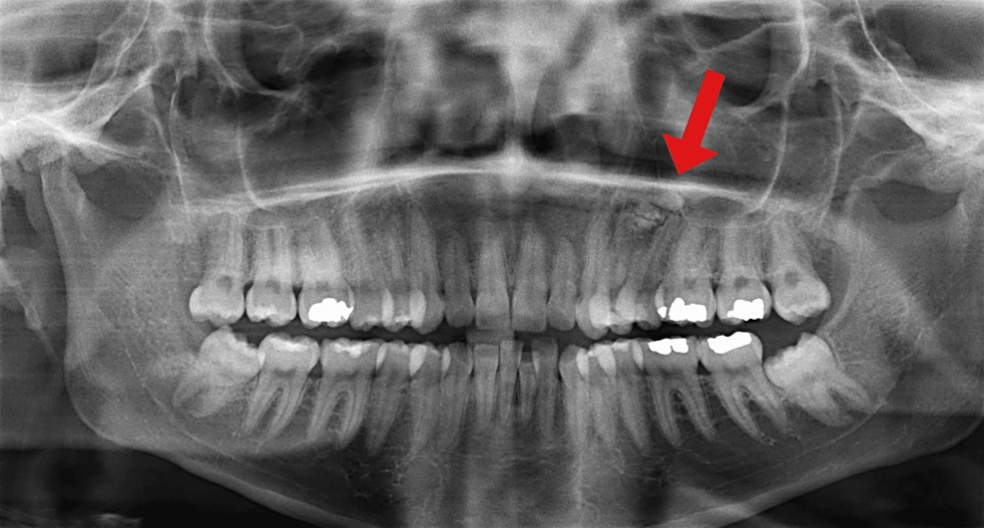
Presentation of the odontoma apical to the first and second premolars on a panoramic radiograph.

**Figure 2:**
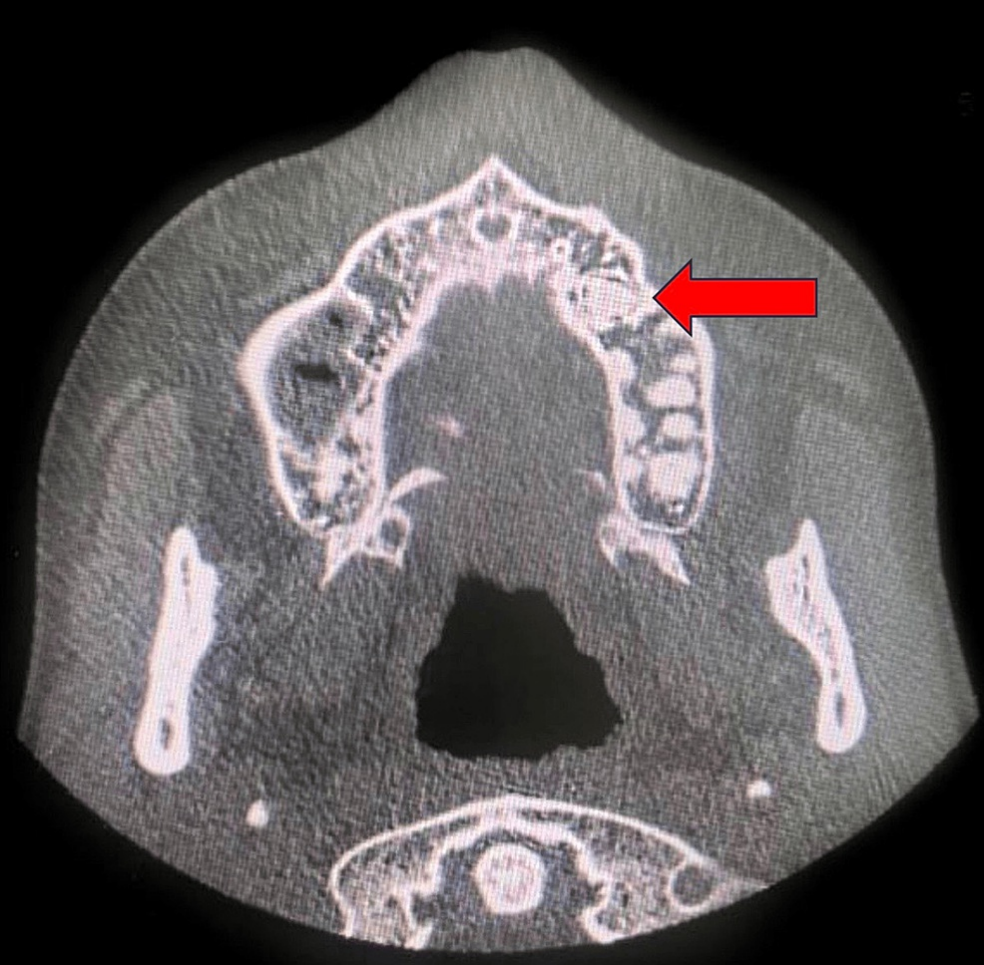
Axial views CT showing a radiopaque mass on the upper left quadrant of the maxilla.

**Figure 3:**
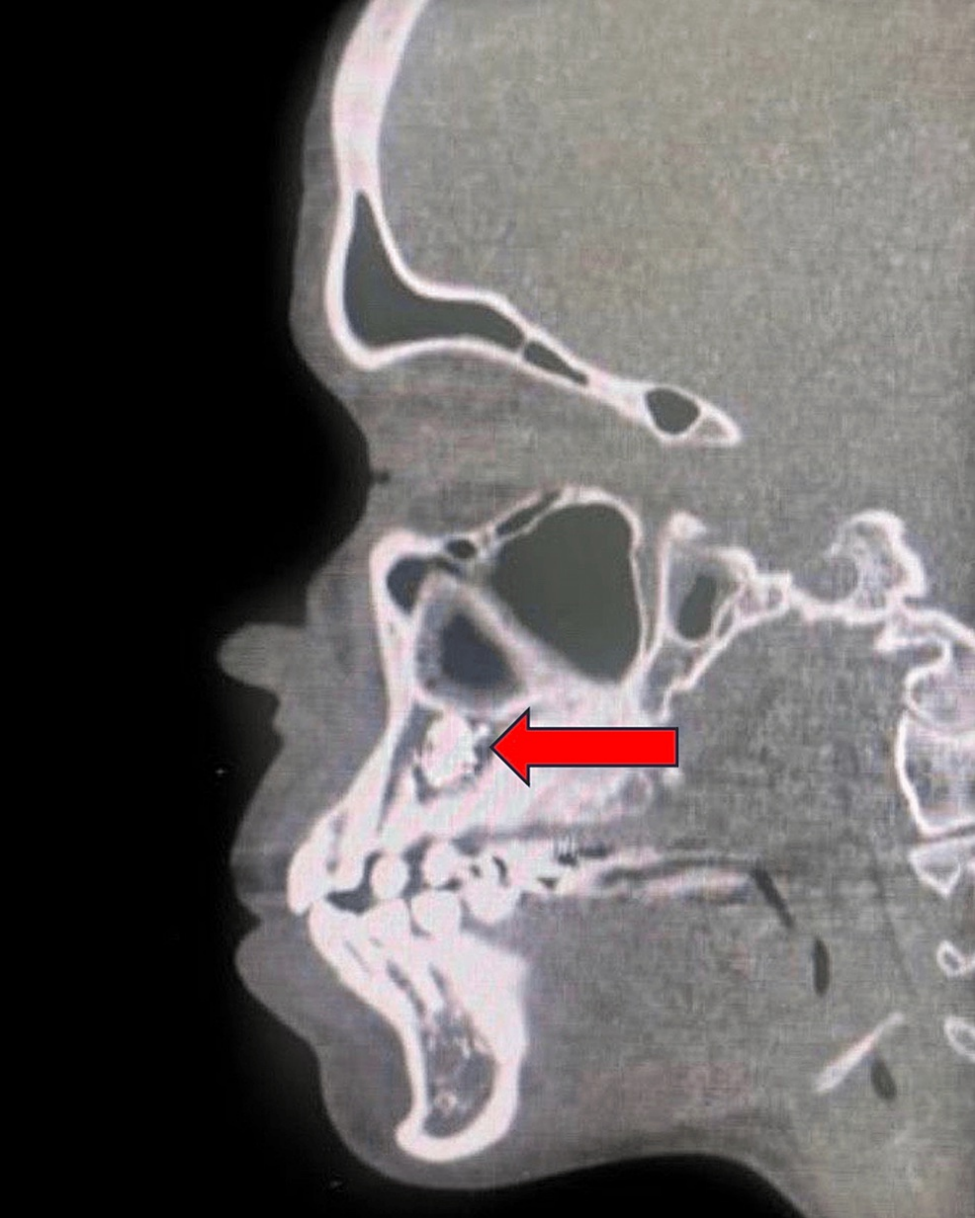
Sagittal view CT showing a radiopaque mass above the premolars in the maxilla.

A 25-year-old female patient with congenital heart disease, severe mitral regurgitation, and NS presented with left maxillary swelling and pain. A radio-opaque mass was detected apically on a panoramic radiograph to the upper left premolar areas (Figure 1). A panoramic radiograph was performed. The findings showed that the left maxillary bone shows a sclerotic lesion with an imaging appearance similar to a distorted tooth, likely representing odontoma near the left upper second premolar tooth. No signs of osseous destruction were observed. Minimal mucosal thickening of the bilateral maxillary sinus was seen. The orbits and visualized parts of the brain appear unremarkable. Axial and sagittal CT views are presented in Figure 2 and Figure 3.

**Figure 4:**
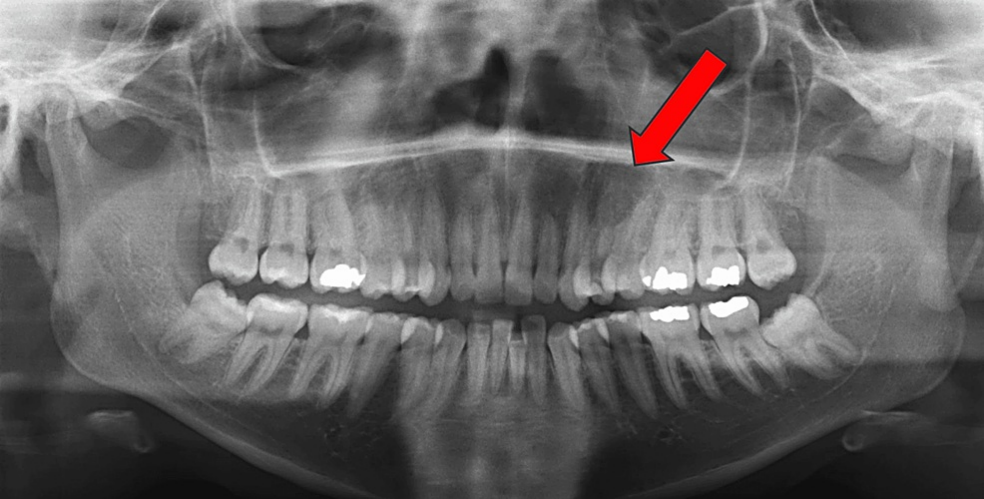
Post-operative panoramic radiograph after complete surgical excision of the left maxillary odontoma.

The patient was shifted to the operating room and intubated nasally. Using 2% lidocaine with 1:100.000 epinephrine as maxillary infiltration, local anesthesia was given. A full-thickness mucoperiosteal flap was raised palatally from #11 to #27, followed by subperiosteal dissection. Once the lesion was identified, it was removed in multiple segments, ensuring enough irrigation. Hemostasis was achieved using suturing with Vicryl 4-0. The patient handled the anesthesia and was extubated in stable condition. Eight hours following the surgical excision of the left maxillary odontoma under GA, the patient was awake, conscious, and oriented with GCS 15/15. The vitals were stable and sating well on room air. Oral intake was good, and the patient was afebrile. Oral examination showed no active bleeding, the wound was not exposed, and the suture was in place. Pain control was established using analgesics. Following one week of surgical excision, the surgical site was intact, with mild swelling, and the suture was in place, with good oral hygiene. Post-operative panoramic radiograph after complete surgical excision of the left maxillary odontoma is shown in Figure 4. 

## Discussion

NS is characterized by various oral manifestations, such as a high-arched palate, misalignment of the teeth, abnormally small jaw, abnormalities in the number of teeth, cysts in the jaw resembling cherubism, severe tooth decay, gum diseases, and large cell lesions [3]. This case report demonstrates a maxillary odontoma in a patient with NS who was fully treated under GA. The oral and maxillofacial manifestations of NS are not yet fully understood. However, many new case reports are being introduced to explain the new manifestations. Lutz et al. explained that conducting an initial dental evaluation and having regular yearly check-ups with oral cleaning as preventive measures is advised. In addition, orthodontics and orthognathic surgery play a crucial role in treating NS patients [1]. Janas et al.'s retrospective study showed that while a comprehensive medical history is generally enough for dental care, this may not hold true for children with NS. Establishing a molecular diagnosis as the standard of care for them is essential. Differentiating between NS and other RASopathies can be difficult, especially in young infants or children. A thorough medical and family history assessment and examination focusing on distinctive features may not be enough. Therefore, it is recommended that these children undergo evaluation by clinical geneticists to interpret gene mutations, enabling appropriate treatment for this patient population [8]. Gürsoy et al. examined the oral, facial, clinical, and molecular traits of individuals with NS [9]. The study found a high frequency of oral and dental issues, such as a high-arched palate, severe dental decay, and gum inflammation, in patients with NS with mutations in the PTPN11 gene.

In addition, it is suggested that the specific mutation, c.181G>A, p.D61N, might be linked to the development of hypodontia in individuals with NS [9]. Uloopi et al presented a case report of unusual dental abnormalities in a child with NS, including multiple permanent teeth that have not erupted, multiple deciduous teeth that are submerged or retained, and supernumerary teeth. These oral findings were linked to other well-known clinical features in the child [10]. Emral et al. demonstrated a case involving a 13-year-old male with NS, who exhibited dental and facial abnormalities. The oral examination revealed a narrow, arched palate, misaligned teeth, and missing teeth from birth. Despite the increased vertical facial pattern observed in cephalometric measurements, a severe deep bite of 9 mm was evident, as described by the author [11]. Odontomas and odontogenic cysts are common in dental practice, but it is uncommon for both lesions to be present in the same area of the body. Furthermore, the diagnosis of these conditions is solely determined through observing the radiographic images [12].

## Conclusions

Overall, this case report points to the association between NS and oral manifestations, explicitly focusing on the rare occurrence of a maxillary odontoma in a patient with NS. The presented patient, who had congenital heart disease and severe mitral regurgitation in addition to NS, developed a left maxillary swelling and pain, prompting further investigation. Radiographic imaging revealed a radio-opaque mass with characteristics suggestive of an odontoma. Given the patient's complex medical history, the surgical excision of the odontoma was performed under GA, with successful outcomes and minimal post-operative complications. While some oral manifestations, such as a high-arched palate, misalignment of teeth, and dental decay, are commonly associated with NS, the occurrence of odontomas in NS patients is less explored, emphasizing the need for further research in this area.

Regular dental evaluations, preventive measures, and appropriate treatment modalities are vital in managing oral and maxillofacial manifestations of NS. In addition, the importance of genetic assessment and diagnosis cannot be understated, especially for differentiating NS from other related syndromes and guiding tailored treatment strategies. As the medical and dental fields continue to advance, more insights into the intricate connections between genetic syndromes and oral health are being uncovered. Collaborative efforts between clinicians and researchers are essential to enhance our understanding of these associations and provide optimal care for individuals with complex conditions, such as NS.
